# Burnout, depression, anxiety and insomnia among medical staff during the COVID-19 epidemic in Shanghai

**DOI:** 10.3389/fpubh.2022.1019635

**Published:** 2023-01-09

**Authors:** Lin Tang, Xin-tong Yu, Yu-wei Wu, Na Zhao, Rui-long Liang, Xiao-lin Gao, Wen-yan Jiang, Yun-fei Chen, Wen-jia Yang

**Affiliations:** ^1^Yueyang Hospital of Integrated Traditional Chinese and Western Medicine, Shanghai University of Traditional Chinese Medicine, Shanghai, China; ^2^Shanghai Fourth People's Hospital Affiliated Tongji University, Shanghai, China

**Keywords:** burnout, anxiety, depression, insomnia, medical staff, COVID-19

## Abstract

**Background:**

Coronavirus disease 2019 (COVID-19) has progressively impacted our daily lives, resulting in unexpected physical and mental stress on medical staff. This study is designed to investigate the levels of and risk factors for burnout, depression, anxiety, and insomnia among medical staff during the COVID-19 epidemic breakout in Shanghai, China.

**Methods:**

This cross-sectional survey was conducted from May 1 to May 31, 2022, among medical staff who were on the frontline during the epidemic breakout in Shanghai from different institutions. The MBI-HSS was used to assess burnout, PHQ-9, GAD-7 and ISI were used to evaluate mental status and insomnia.

**Results:**

A total of 543 valid questionnaires were collected. The depersonalization, depression, anxiety, and insomnia scores of medical staff were significantly higher during the pandemic in Shanghai compared with norms, while lack of personal achievement scores were decreased. Working time, work unit, work environment and age are important influencers of burnout, depression and anxiety of medical staff. Long working hours are the most likely causes of burnout and emotional disorders. Medical staff in primary hospitals were most likely to suffer from burnout and emotional disorders, while medical staff in tertiary hospitals had a reduced sense of personal achievement. Young medical staff are prone to negative emotions such as depression and anxiety, while older medical staff have a lower sense of personal accomplishment. Medical staff who were not in the shelter hospitals or designated hospitals were more likely to have problems of emotional exhaustion, depersonalization and anxiety than those who were in the shelter hospitals or designated hospitals. Contracting COVID-19 had no effect on medical staff. Emotional exhaustion and depersonalization were positively correlated with anxiety, depression, and sleep disorders while personal achievement was negatively correlated with these factors.

**Conclusion:**

Medical staff in Shanghai had high burnout, depression, anxiety and insomnia levels during the epidemic outbreak in Shanghai. During the COVID-19, medical staff may suffer different psychological problems which should be concerned. Care and supports about burnout, mental health and insomnia need to be taken to promote the mental health of medical staff according to different characteristics of medical staff.

## Introduction

Coronavirus disease 2019 (COVID-19) has impacted the economic, social, behavioral, and medical aspects of our lives (1–3). The WHO declared the 2019-nCoV pandemic a public health emergency of international concern on January 30, 2020. The disease was officially named coronavirus disease 2019 (COVID-19) on February 11, 2020 (4). Globally, over 514 million confirmed cases of severe acute respiratory syndrome coronavirus 2 (SARS-CoV-2) have been reported, resulting in over six million deaths (11 May 2022) (5). As of February 17th 2020, 3019 medical personnel were affected by SARS-CoV-2 on mainland China, of whom 1, 716 had confirmed cases (6). In late February 2022, a wave of SARS-CoV-2 infections rapidly appeared in Shanghai, China. According to the Shanghai Municipal Health Commission, 649, 341 cases were identified as of May 31, 2022, including 591, 341 asymptomatic carriers. In order to actually reduce the number of people infected by COVID-19, permit the early diagnosis and appropriate treatment of severe COVID-19, and thereby minimizing the case fatality rate, strict and comprehensive pandemic control strategies were implemented in Shanghai (7). All medical staff, including those from other provinces, were involved in epidemic prevention. During the COVID-19 pandemic, medical staff were under great work intensity and psychological stress. Mental disorders and illnesses, especially increased anxiety reactions, depressive symptoms, and stress became more prevalent among medical staff (8). The possibility of mandating medical personnel to fight the pandemic in a different workplace and organizational changes involving the transformation of hospitals or departments into COVID units caused fear and disapproval among staff. Contact with infected patients greatly increased the risk of infection, especially among frontline healthcare workers working under stressful conditions within the medical emergency system during the COVID-19 pandemic. These conditions have been shown to be associated with severe mental health problems among frontline medical staff (9). Frontline medical staff refer to the medical staff who directly contact with the coronavirus like the staff worked in the shelter hospital or participated in the sampling in community. Medical staff also experienced psychological stress from work-related burnout and somatic symptoms (10). A meta-analysis of the awareness rate of medical staff shows that the highest awareness rate comes from psychological hospital staff, with an awareness rate of 88%; the awareness rate of non-psychiatric medical staff was only 68%, which was lower than general population (11). Meanwhile, another meta-analysis shows key point studies that conducted in China reported more mental problems than in other countries (12). Therefore, it is very important to investigate the mental health of frontline medical staff during the epidemic.

The concept of job burnout was first put forward by Freudenberger in 1996. Burnout is used to describe mental, emotional, and physical stress due to three dimensions of occupational stressors: emotional exhaustion, depersonalization, and personal achievement (13). Job burnout was officially classified as an occupational health syndrome in the latest World Health Organization International Classification of Diseases (ICD-11) (14). Huo found that job burnout of medical staff during the COVID-19 epidemic was positively correlated with depression symptoms, which were in turn related to increased occupational stress during the epidemic (15). Other studies found that job burnout led to negative emotions among medical staff, had a negative impact on work quality, was associated with deterioration in medical service quality and tensions between medical staff and patients, and could even result in hidden dangers to medical safety (16, 17). Understanding burnout among medical staff during the epidemic is therefore important to ensuring health of medical staff and patient safety.

The aims of this study were: (1) investigate the status of burnout, depression, anxiety and insomnia among medical staff during the COVID-19 epidemic outbreak in Shanghai; (2) explore factors related to burnout, anxiety, depression, and sleep disorder, and give suggestion and potentially helpful methods; (3) collect burnout, mental health and sleep quality data of medical staff during the epidemic in Shanghai, which could provide the basis for constructing future interventions during large-scale medical emergencies.

## Methods

### Study design and participants

This study was a cross-sectional survey. Subjects were recruited *via* questionnaires from May 1 to May 31, 2022. The questionnaire was distributed to several units using the online mini-program “Wenjuanxing” (WJX, Changsha, China). We set specific personnel to deliver questionnaires in 5 shelter hospitals, 5 tertiary hospitals, 5 secondary hospitals and 5 basic hospitals from most administrative regions in Shanghai to ensure that the subjects are the target population of the study. Meanwhile the specific personnel in each unit explained and guided the participants to fill in the questionnaire, so the quality of the returned questionnaire can be ensured. Participants were informed that if the survey was completed, an informed consent form would be signed by default. The survey was set so that the same IP address could only fill out the questionnaire once.

**Figure 1:**
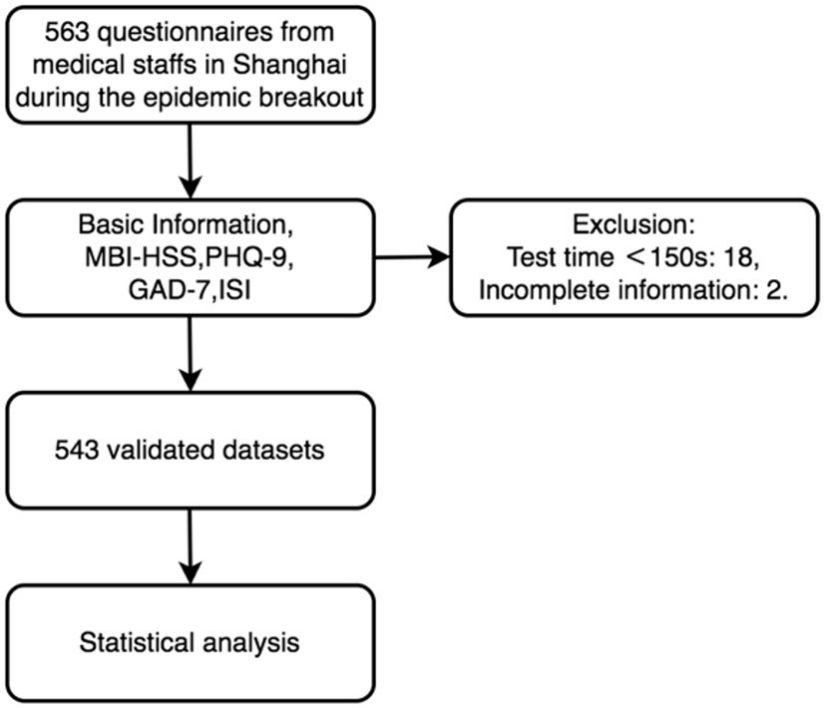
Study participant selection flow chart.

The inclusion criteria were: (1) age between 18 and 60, and (2) medical staff working in Shanghai during the COVID-19 epidemic outbreak. Individuals who failed to complete the whole survey or whose answer time was <150 s were excluded. The flow chart for this study is shown in Figure 1.

### Outcome measures

#### Demographics

The first part of the survey included basic information such as age, gender, marital status, number of children, daily work time and job category.

#### Assessment scales

We chose The Maslach Burnout Inventory-Human Services Survey (MBI-HSS) to evaluate the burnout level of participants. The MBI-HSS is a self-reported questionnaire with a total of 22 items that is used to evaluate the burnout of service personnel including medical staff (18). The sub-scales of the questionnaire are: emotional exhaustion (EE, 9 items), depersonalization (DE, 5 items) and lack of personal achievement (PA, 8 items). A study showed the test-retest reliability or reproducibility showed an Intraclass Correlation Coefficient of 0.95. The internal consistency of the survey was 0.922. The cut-off point for the existence of burnout achieved a sensitivity of 92.2%, a specificity of 92.1% (19), which means this questionnaire was found to be viable, valid, and reliable for measuring burnout.

A meta-analysis about the impact of the COVID-19 on mental health of medical staff found the depression, anxiety and insomnia symptoms representing the most robust evidence based on a large dataset of prevalence meta-analyses (20). So, we chose the Patient Health Questionnaire (PHQ-9), the Generalized Anxiety Disorder Scale (GAD-7) and the Insomnia Severity Index (ISI) for assessment of depression, anxiety and insomnia symptoms.

The patient health questionnaire (PHQ-9) is used to assess an individual's depressive symptoms over the prior 2 weeks and is a widely used depression screening tool. Research have proved the validity and reliability of PHQ-9 for different kinds of people, internal consistency of the PHQ-9 was high (α = 0.87) (21), when the total PHQ-9 score was used to identify depression, the Area under the Curve (AUC) was 0.93 (95% confidence interval [CI], 0.88–0.97) on Receiver Operating Characteristic (ROC) analysis (22).

The generalized anxiety disorder scale (GAD-7) is used to assess anxiety symptoms over the past 2 weeks. A study for screening the anxiety among pregnant Chinese women found the sensitivity of GAD-7 is 96.8%, and the specificity is 56.1% which proved the quality of GAD-7. Other studies also showed that GAD-7 is capable of detecting symptoms and generalized anxiety disorder, and it is a useful and simple tool for detecting anxiety-related problems (23, 24).

The insomnia severity index (ISI) is a brief screening measure for insomnia that evaluates sleep related conditions over the past 2 weeks. ISI is a brief, reliable, and valid instrument to facilitate screening for insomnia in general practice. The internal consistency of ISI was excellent (Cronbach α = 0.92), and the cutoff score of 14 was optimal (82.4% sensitivity, 82.1% specificity, and 82.2% agreement) in a study in primary care, (25) there are other studies verified the value of clinical use of ISI in different language (26–28). All the detailed score category has been listed in Table 1.

**Table 1:** The severity levels of the scales.

**Scales**	**No Symptom**	**Mild**	**Moderate**	**Severe**
**MBI-total score** (29)	0–28	29–43	44–87	>88
**MBI-EE** (30)	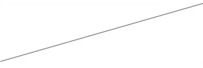	0–16	17–26	27–54
**MBI-DE** (30)	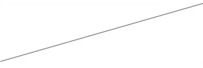	0–6	7–12	13–30
**MBI-PA** (30)	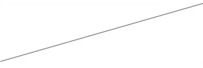	0–31	32–38	39–43
**PHQ-9** (31)	<5	5–9	10–14	>15
**GAD-7** (31)	<5	5–9	10–14	>15
**ISI** (32)	<8	8–14	15–21	22–28

#### Sample size calculation

Estimates suggest that 48.6% of the population of physicians who work in hospitals develop burnout symptoms (33). In this study, considering the influence of random sampling, we set the design efficiency (DF) to 1.2 to appropriately expand the sample size. We made δ = 0.1 p and α = 0.05 (two-side) according to the Epidemiology (34). According to the formula


$$\begin{array}{l} N=DF\times \frac{U^{2}\times p\left(1-p\right)}{\delta^{2}} \end{array}$$

we calculated *n* = 488. Considering a loss factor of 10%, the final sample size was 537. Our sample was larger than this, permitting the survey to not only explore the level and risk factors for burnout syndrome, depression, anxiety, and insomnia, but to also investigate the association between burnout and depression, anxiety, and sleep disorders among medical staff during the COVID-19 epidemic breakout in Shanghai.

### Statistical analysis

SPSS 25.0 and the open source software R 4.0 (Ross Ihaka, Robert Gentleman, New Zealand) (35) were used for analysis. Cronbach's alpha was used to assess the reliability of the anxiety scale. Enumeration data were described by the number of cases and percentages. The sample size reached 543, and the measurement data approximately met a normal distribution. A large-sample Z test was used for comparisons with the norm, a two independent samples *t*-test was used for comparisons between two groups, and comparisons between multiple groups were performed using one-way ANOVA with the LSD' *t*-test for multiple between-group comparisons.

All variables with *p* < 0.10 in the univariate analyses were included in multivariate stepwise regression models to identify independent influencing factors. The inspection level α was set as 0.05.

## Results

### Demographic characteristics

A total of 563 questionnaires were collected in this study of which and 543 were valid, yielding proportion of data that can be analyzed is 96.45%. Of the included participants, 388 (71.45%) were female and 155 (8.55%) were male, and 278 (51.20%) worked at the designated hospital specified for patients affected by SARS-CoV-2. More detailed information about the demographics and job-related characteristics of the included participants is shown in Table 2. Cronbach reliability analysis was used to verify the reliability of MBI-HSS, PHQ-9, GAD-7 and ISI. The Cronbach α coefficients of MBI-HSS, PHQ-9, GAD-7 and ISI were, respectively, 0.773, 0.894, 0.942, and 0.925, indicating that their reliabilities were high. The KMO and Bartlett tests were used to verify the validity. The KMO values of MBI-HSS, PHQ-9, GAD-7 and ISI were respectively 0.929, 0.898, 0.927, and 0.903, indicating that our data was very suitable for extracting information, and its validity was good.

**Table 2:** Demographic data of participants ( *n* = 543).

**Variable name**	**Description**
**Gender**	
Male	155 (28.55)
Female	388 (71.45)
**Age (years old)**	
≤25	42 (7.73)
26–35	241 (44.38)
36–45	168 (30.94)
>45	92 (16.94)
**Length of service (years)**	
≤5	126 (23.20)
6–10	128 (23.57)
11–15	114 (20.99)
>15	175 (32.23)
**Education level**	
Junior college education	54 (9.94)
Bachelor	238 (43.83)
Master	173 (31.86)
Doctor	78 (14.36)
**Professional title grade**	
None	38 (7.00)
Junior	161 (29.65)
Middle rank	230 (42.36)
Senior	114 (20.99)
**Marital status**	
Single	166 (30.57)
Married	377 (69.43)
**Numbers of children**	
Zero	213 (39.23)
One	273 (50.28)
More than one	57 (10.50)
**Hospital level**	
Basic hospital (community)	58 (11.15)
Secondary hospital	79 (15.19)
Tertiary hospital	383 (73.65)
**Job category**	
Administrative staff	29 (5.34)
Medical technicians	86 (15.84)
Nurses	155 (28.55)
Clinicians	243 (44.75)
Others	30 (5.52)
**Daily working time (hours)**	
<9	327 (60.22)
9–10	135 (24.86)
10–11	33 (6.08)
>11	48 (8.84)
**Experience of working in shelter hospital or**	
**designated hospital for patients affected by COVID-19**	
No	265 (48.80)
Yes	278 (51.20)
**Medical staff from other provinces to help Shanghai?**	
No	471 (86.74)
Yes	72 (13.26)
**Experience of affected by COVID-19**	
No	518 (95.40)
Yes	25 (4.60)
PHQ-9	8.31 ± 5.17
GAD-7	6.65 ± 4.89
ISI	9.95 ± 6.48
MBI-EE	23.09 ± 9.24
MBI-DE	7.97 ± 4.82
MBI-PA	24.74 ± 6.62

### Burnout, anxiety, depression, and sleep disorder scores during COVID-19 compared to norms

We compared burnout, anxiety, depression, and sleep disorder scores of Shanghai medical staff in epidemic in 2022 with the pre-epidemic norms. As indicated in Table 3, MBI-PA scores were significantly lower and MBI-DE scores significantly higher during the pandemic compared with pre-epidemic norms (*P* < 0.01) (36), MBI-EE scores were not statistically different from pre-epidemic norms (*P* > 0.05) (36). Depression, anxiety, and sleep disorder scores among the medical staff in Shanghai were significantly higher during the pandemic compared with before (*P* < 0.01) (37, 38). These results mean that Shanghai medical workers experienced job burnout during the epidemic, with their mood and sleep affected, but their lack of personal achievement decreased.

**Table 3:** Comparison of job burnout, mental health and sleep status of medical workers in Shanghai with the norm (x ± s, *n* = 543).

**Variable**	**Shanghai medical staff in epidemic in 2022 (*N* = 543)**	**Medical staff(norm)**		**Difference estimation (95%)**	***z***	***p***
	**Point**	***N***	**Point**			
PHQ-9	8.31 ± 5.17	761	6.33 ± 5.59	1.980 (1.391, 2.569)	6.689	<0.001
GAD-7	6.65 ± 4.89	761	4.75 ± 4.54	1.900 (1.377, 2.423)	7.124	<0.001
ISI	9.95 ± 6.48	927	6.70 ± 5.76	3.250 (2.591, 3.909)	9.663	<0.001
MBI-EE	23.09 ± 9.24	1,320	23.02 ± 10.29	0.040 (-0.915, 0.995)	0.082	0.935
MBI-DE	7.97 ± 4.82	1,320	6.81 ± 5.57	1.160 (0.655, 1.665)	4.505	<0.001
MBI-PA	24.74 ± 6.62	1,320	28.55 ± 9.34	−3.810 (−4.561, −3.059)	−9.944	<0.001

### Burnout and associated factors

As shown in Table 4, univariate analysis associated emotional exhaustion with age, hospital level, job category, daily working time, experience with working in a shelter hospital or a designated hospital for patients affected by COVID-19, and whether they were came from other provinces to help Shanghai (*P* < 0.05). Depersonalization was related to professional title grade, marital status, number of children, hospital level, job category, daily working time, whether they had experience working in a shelter hospital or a designated hospital for patients affected by COVID-19, and whether they came from other provinces to help Shanghai (*P* < 0.05). Lack of personal accomplishment was related to gender, age, length of service, educational level, professional title grade, marital status, number of children, and hospital level (*P* < 0.05). Being infected with COVID-19 had no effect on burnout among medical staff (*P* > 0.05) (Table 4).

**Table 4:** Single factor analysis of job burnout, mental health and sleep status of medical workers in Shanghai.

	**N**	**PHQ-9**	**GAD-7**	**ISI**	**MBI-EE**	**MBI-DE**	**MBI-PA**
**Gender**							^****^
Male	155	8.57 ± 5.79	6.59 ± 4.97	9.85 ± 6.42	23.21 ± 9.37	8.23 ± 4.98	26.34 ± 6.91
Female	388	8.21 ± 4.91	6.67 ± 4.87	9.99 ± 6.51	23.04 ± 9.21	7.86 ± 4.75	24.11 ± 6.41
Age (years old)		^*^	^*^		^**^		^****^
≤25	42	8.86 ± 5.91	6.74 ± 5.26	10.48 ± 6.60	21.83 ± 10.15^b^	7.95 ± 4.75	22.50 ± 7.73^b^
26–35	241	8.09 ± 5.12	6.69 ± 4.80	9.46 ± 6.20	22.00 ± 9.49^b^	8.25 ± 5.18	24.47 ± 6.48^b^
36–45	168	9.01 ± 5.35	7.18 ± 5.13	10.65 ± 6.85	24.42 ± 9.03^a^	8.01 ± 4.72	24.29 ± 6.19^b^
>45	92	7.38 ± 4.47	5.53 ± 4.36	9.71 ± 6.42	24.09 ± 8.19^ab^	7.14 ± 3.93	27.32 ± 6.62^a^
**Length of service (years)**							^***^
≤5	126	8.37 ± 5.22	6.85 ± 4.78	9.35 ± 6.33	22.07 ± 9.25	8.07 ± 4.63	23.07 ± 6.54^c^
6–10	128	8.53 ± 5.35	7.03 ± 5.12	9.73 ± 6.26	22.37 ± 9.45	8.47 ± 5.35	24.45 ± 6.39^bc^
11–15	114	8.06 ± 5.29	6.54 ± 5.10	10.31 ± 6.71	23.07 ± 10.11	7.72 ± 5.17	24.79 ± 6.49^ab^
>15	175	8.29 ± 4.96	6.30 ± 4.67	10.31 ± 6.60	24.37 ± 8.39	7.68 ± 4.28	26.13 ± 6.70^a^
**Education level**							^**^
Junior college education	54	7.28 ± 4.41	5.52 ± 4.21	9.93 ± 6.32	20.61 ± 8.44	7.00 ± 4.24	22.98 ± 6.22^c^
Bachelor	238	8.37 ± 5.67	6.50 ± 5.14	10.38 ± 6.97	22.84 ± 10.01	8.16 ± 5.17	25.20 ± 7.29^ab^
Master	173	8.62 ± 4.88	7.23 ± 4.83	9.71 ± 6.15	23.68 ± 8.67	8.23 ± 4.71	24.05 ± 5.80^bc^
Doctor	78	8.17 ± 4.67	6.60 ± 4.61	9.18 ± 5.72	24.28 ± 8.35	7.47 ± 4.20	26.12 ± 6.13^a^
Professional title grade				^*^		^**^	^***^
None	38	8.37 ± 5.96	6.61 ± 5.03	9.24 ± 6.31	22.92 ± 10.08	8.37 ± 4.31^ab^	22.55 ± 6.80^b^
Junior	161	8.25 ± 5.30	6.83 ± 4.91	10.00 ± 6.59	22.09 ± 10.01	7.90 ± 5.15^ab^	24.03 ± 7.02^b^
Middle rank	230	8.80 ± 5.35	6.98 ± 5.13	10.66 ± 6.59	23.60 ± 9.18	8.50 ± 5.11^a^	24.69 ± 6.29^b^
Senior	114	7.41 ± 4.18	5.75 ± 4.24	8.68 ± 6.01	23.54 ± 7.86	6.83 ± 3.57^b^	26.59 ± 6.30^a^
Marital status		^*^				^***^	^***^
Single	166	8.90 ± 5.38	7.10 ± 4.98	10.34 ± 6.02	23.48 ± 8.97	8.84 ± 4.91	23.51 ± 6.23
Married	377	8.06 ± 5.06	6.45 ± 4.84	9.77 ± 6.67	22.92 ± 9.37	7.58 ± 4.73	25.29 ± 6.73
Numbers of children		^*^			^*^	^***^	^***^
Zero	213	8.63 ± 5.09	6.83 ± 4.73	9.81 ± 6.11	22.92 ± 9.03	8.78 ± 4.79^a^	23.54 ± 6.18^b^
One	273	8.36 ± 5.39	6.64 ± 5.01	10.25 ± 6.95	23.72 ± 9.16	7.68 ± 4.75^b^	25.50 ± 6.77^a^
More than one	57	6.93 ± 4.16	6.00 ± 4.94	9.02 ± 5.40	20.72 ± 10.14	6.28 ± 4.72^c^	25.63 ± 6.98^a^
Hospital level		^****^	^****^	^**^	^****^	^***^	^**^
Basic hospital (community)	58	9.64 ± 6.09^a^	6.83 ± 5.57^b^	10.84 ± 6.76^ab^	25.21 ± 9.11^a^	9.62 ± 5.01^a^	23.40 ± 6.30^b^
Secondary hospital	79	10.47 ± 5.98^a^	8.62 ± 5.38^a^	11.35 ± 6.92^a^	26.30 ± 10.40^a^	9.16 ± 5.12^a^	23.48 ± 5.88^b^
Tertiary hospital	383	7.64 ± 4.63^b^	6.21 ± 4.60^b^	9.58 ± 6.35^b^	22.24 ± 8.84^b^	7.52 ± 4.64^b^	25.15 ± 6.84^a^
Job category		^*^	^**^		^**^	^**^	
Administrative staff	29	8.07 ± 4.35	6.45 ± 4.98^ab^	9.07 ± 5.86	22.66 ± 8.84^ab^	8.24 ± 4.25^ab^	24.83 ± 6.24
Medical technicians	86	9.06 ± 5.54	7.52 ± 5.26^a^	10.10 ± 6.81	23.95 ± 9.79^a^	8.92 ± 5.25^a^	24.98 ± 6.73
Nurses	155	7.31 ± 4.83	5.61 ± 4.61^b^	10.39 ± 6.82	20.91 ± 9.54^ab^	7.03 ± 4.78^b^	24.70 ± 7.17
Clinicians	243	8.62 ± 5.20	6.89 ± 4.83^a^	9.69 ± 6.29	24.09 ± 8.84^a^	8.22 ± 4.73^ab^	24.73 ± 6.23
Others	30	9.13 ± 5.81	7.73 ± 5.01^a^	10.13 ± 6.06	24.20 ± 8.26^a^	7.77 ± 4.38^ab^	24.37 ± 7.19
Daily working time (hours)		^**^	^*^		^****^	^***^	
<9	327	7.78 ± 4.82^b^	6.20 ± 4.55	9.51 ± 6.31	21.19 ± 8.88^c^	7.40 ± 4.63^b^	24.96 ± 6.72
9–10	135	9.33 ± 5.99^a^	7.40 ± 5.57	10.34 ± 6.70	25.15 ± 9.27^b^	8.74 ± 5.09^a^	23.82 ± 6.33
10–11	33	8.64 ± 5.25^ab^	6.73 ± 4.35	10.36 ± 6.79	24.94 ± 8.32^b^	8.48 ± 4.49^ab^	24.91 ± 6.34
>11	48	8.88 ± 4.55^ab^	7.58 ± 5.18	11.56 ± 6.64	29.02 ± 8.38^a^	9.25 ± 5.01^a^	25.73 ± 6.84
Experience of working in shelter hospital or designated hospital for patients affected by COVID-19		^**^	^***^		^****^	^***^	
No	265	8.82 ± 5.31	7.22 ± 5.04	9.82 ± 6.46	24.65 ± 8.83	8.68 ± 4.82	24.59 ± 6.36
Yes	278	7.83 ± 4.99	6.11 ± 4.69	10.08 ± 6.51	21.60 ± 9.40	7.29 ± 4.72	24.89 ± 6.87
Medical staff from other provinces to help Shanghai?		^****^	^****^		^****^	^***^	
No	471	8.65 ± 5.17	6.96 ± 4.88	10.12 ± 6.45	23.71 ± 8.82	8.20 ± 4.71	24.72 ± 6.38
Yes	72	6.10 ± 4.64	4.65 ± 4.48	8.82 ± 6.59	19.06 ± 10.88	6.44 ± 5.26	24.93 ± 8.07
**Experience of affected by COVID-19**							
No	518	8.31 ± 5.19	6.59 ± 4.87	9.98 ± 6.52	23.06 ± 9.33	7.92 ± 4.84	24.80 ± 6.62
Yes	25	8.32 ± 4.85	7.92 ± 5.20	9.24 ± 5.78	23.72 ± 7.27	9.00 ± 4.19	23.60 ± 6.66

1. ^*^P < 0.1, ^**^P < 0.05, ^***^P < 0.01, ^****^P < 0.001; 2. There was no significant difference between the two groups with the same letter.

All variables with *P* < 0.10 in the univariate analyses (Table 4) were included in the multivariate stepwise regression (Table 5), which showed the impact of each of the selected background factors on burnout when controlling for all other factors. Medical staff who were working for long time were more likely to have emotional exhaustion (*P* < 0.01). Medical staff who were not working in a shelter hospital or a designated hospital for patients affected by COVID-19 were more likely to have emotional exhaustion than those who were (*P* < 0.01).

**Table 5:** Multivariate analysis of job burnout, mental health and sleep status of medical workers in Shanghai.

	**Variable**	**Estimate**	**Se**	***t***	***p***	**VIF**
MBI-EE	(Intercept)	24.019 (21.421, 26.616)	1.322	18.167	<0.001	1.084
**Hospital level**						
	Basic hospital (community)	Ref.				
	Secondary hospital	1.746 (−1.232, 4.723)	1.516	1.152	0.25	
	Tertiary hospital	−1.511 (−3.989, 0.968)	1.261	−1.198	0.232	1.089
**Daily working time (hours)**						
	<9	Ref.				
	9–10	3.293 (1.475, 5.112)	0.926	3.558	<0.001	
	10–11	2.681 (−0.529, 5.89)	1.634	1.641	0.101	
	>11	6.279 (3.45, 9.108)	1.44	4.361	<0.001	1.176
**Experience of working in shelter hospital or designated hospital for patients affected by COVID-19**						
	No	Ref.				
	Yes	−2.229 (−3.864, −0.595)	0.832	−2.68	0.008	1.182
**Medical staff from other provinces to help Shanghai?**						
	No	Ref.				
	Yes	−2.365 (−4.747, 0.016)	1.212	−1.951	0.052	
MBI-DE	(Intercept)	10.655 (8.618, 12.693)	1.037	10.275	<0.001	
	Professional title grade					1.53
	None	Ref.				
	Junior	0.068 (−1.666, 1.803)	0.883	0.077	0.938	
	Middle rank	0.684 (−1.098, 2.465)	0.907	0.754	0.451	
	Senior	−1.33 (−3.302, 0.643)	1.004	−1.324	0.186	
	Numbers of children					1.397
	Zero	Ref.				
	One	−1.242 (−2.216, −0.269)	0.496	−2.507	0.012	
	More than one	−2.07 (−3.542, −0.598)	0.749	−2.764	0.006	
	Hospital level					1.07
	Basic hospital (community)	Ref.				
	Secondary hospital	−0.509 (−2.077, 1.06)	0.798	−0.637	0.524	
	Tertiary hospital	−1.873 (−3.168, −0.578)	0.659	−2.842	0.005	
	Daily working time (hours)					1.204
	<9	Ref.				
	9–10	1.211 (0.232, 2.19)	0.498	2.431	0.015	
	10–11	1.033 (−0.682, 2.747)	0.873	1.184	0.237	
	>11	1.425 (−0.088, 2.939)	0.77	1.85	0.065	
	Experience of working in shelter hospital or designated hospital for patients affected by COVID-19					1.081
	No	Ref.				
	Yes	−1.653 (−2.476, −0.831)	0.419	−3.948	<0.001	
MBI-PA	(Intercept)	21.944 (19.033, 24.856)	1.482	14.807	<0.001	
	Gender					1.042
	Male	Ref.				
	Female	−1.797 (−3.069, −0.525)	0.647	−2.775	0.006	
	Age (years old)					1.059
	≤25	Ref.				
	26–35	2.4 (0.182, 4.618)	1.129	2.126	0.034	
	36–45	1.989 (−0.305, 4.284)	1.168	1.703	0.089	
	>45	4.622 (2.11, 7.135)	1.279	3.615	<0.001	
	Hospital level					1.018
	Basic hospital (community)	Ref.				
	Secondary hospital	0.356 (−1.859, 2.571)	1.128	0.315	0.753	
	Tertiary hospital	2.09 (0.279, 3.901)	0.922	2.267	0.024	
PHQ-9	(Intercept)	10.471 (8.367, 12.574)	1.071	9.781	<0.001	
	Age (years old)					1.160
	≤25	Ref.				
	26–35	−1.327 (−3.023, 0.37)	0.864	−1.536	0.125	
	36–45	−0.972 (−2.754, 0.811)	0.907	−1.071	0.285	
	>45	−2.484 (−4.439, −0.529)	0.995	−2.496	0.013	
	Hospital level					1.080
	Basic hospital (community)	Ref.				
	Secondary hospital	0.843 (−0.849, 2.534)	0.861	0.978	0.328	
	Tertiary hospital	−1.507 (−2.914, −0.1)	0.716	−2.104	0.036	
	Medical staff from other provinces to help Shanghai?					1.113
	No	Ref.				
	Yes	−2.309 (−3.619,−0.998)	0.667	−3.460	0.001	
	Daily working time (hours)					1.128
	<9	Ref.				
	9–10	1.316 (0.275, 2.356)	0.529	2.485	0.013	
	10–11	0.740 (−1.094, 2.575)	0.934	0.793	0.428	
	>11	0.586 (−1.032, 2.203)	0.823	0.712	0.477	
GAD-7	(Intercept)	8.302 (6.255, 10.35)	1.042	7.967	<0.001	
	Age (years old)					1.127
	≤25	Ref.				
	26–35	−0.583 (−2.21, 1.044)	0.828	−0.704	0.482	
	36-45	−0.416 (−2.112, 1.279)	0.863	−0.482	0.63	
	>45	−2.257 (−4.126, −0.388)	0.952	−2.372	0.018	
	Hospital level					1.055
	Basic hospital (community)	Ref.				
	Secondary hospital	1.803 (0.184, 3.421)	0.824	2.188	0.029	
	Tertiary hospital	−0.424 (−1.76, 0.912)	0.68	−0.623	0.534	
	Experience of working in shelter hospital or designated hospital for patients affected by COVID-19					1.182
	No	Ref.				
	Yes	−1.15 (−2.042, −0.259)	0.454	−2.535	0.012	
	Medical staff from other provinces to help Shanghai?					1.231
	No					
	Yes	−1.811 (−3.133, −0.489)	0.673	−2.692	0.007	
ISI	(Intercept)	10.633 (7.869, 13.398)	1.407	7.557	<0.001	
	Professional title grade					1.025
	None	Ref.				
	Junior	0.257 (−2.134, 2.647)	1.217	0.211	0.833	
	Middle rank	0.899 (−1.425, 3.222)	1.183	0.76	0.448	
	Senior	−0.977 (−3.465, 1.511)	1.267	−0.772	0.441	
	Hospital level					1.025
	Basic hospital (community)					
	Secondary hospital	0.349 (−1.856, 2.554)	1.123	0.311	0.756	
	Tertiary hospital	−1.308 (−3.113, 0.496)	0.919	−1.424	0.155	

Medical staff without children were more likely to have depersonalization than those with children (*P* < 0.05). Medical staff of basic hospitals were more likely to have depersonalization than those of tertiary hospitals (*P* < 0.01). Medical staff who were working for long time were more likely to have depersonalization (*P* < 0.05). Medical staff who were not working in a shelter hospital or a designated hospital for patients affected by COVID-19 were more likely to have depersonalization than those who were (*P* < 0.01). Male medical staff, old medical staff were more likely to feel a lack of personal accomplishment (*P* < 0.05). Medical staff of tertiary hospitals were more likely to feel a lack of personal accomplishment than those of basic hospitals (*P* < 0.05) (Table 5).

### Depression and associated factors

As shown in Table 4, the univariate analysis found that depression among the medical staff was related to the hospital level, daily working time, experience working in a shelter hospital or a designated hospital for patients affected by COVID-19, and whether they came from other provinces to help Shanghai (*P* < 0.05). Being infected with COVID-19 had no effect on depression among medical staff (*P* > 0.05) (Table 4).

Young medical staff were more likely to be depressed than old ones (*P* < 0.05). Medical staff of basic hospitals were more likely to be depressed than those of tertiary hospitals (*P* < 0.05). Medical staff who were working for long time were more likely to be depressed (*P* < 0.05). Medical staff from other provinces to help Shanghai were less likely to have depression than those of Shanghai (*P* < 0.01) (Table 5).

### Anxiety and associated factors

As shown in Table 4, univariate analyses showed that anxiety among medical staff was related to hospital level, job category, experience working in a shelter hospital or a designated hospital for patients affected by COVID-19, and whether they came from other provinces to help Shanghai (*P* < 0.05). Being infected with COVID-19 had no effect on anxiety among medical staff (*P* > 0.05) (Table 4).

The multivariable analysis (Table 5) identified young medical staff were more likely to have anxiety than old ones (*P* < 0.05). Medical staff of secondary hospitals were more likely to have anxiety than those of basic hospitals (*P* < 0.05). Medical staff who were not working in a shelter hospital or a designated hospital for patients affected by COVID-19 were more likely to have anxiety than those who were (*P* < 0.05). Medical staff from other provinces to help Shanghai were less likely to have anxiety than those of Shanghai (*P* < 0.01) (Table 5).

### Sleep disorder and associated factors

As shown in Table 4, univariate analysis found sleep disorders among the medical staff were only related to the hospital level (*P* < 0.05). The multivariable analysis found no statistically significant difference in the ISI scores of medical staff with different work units (*P* > 0.05) (Table 5).

### The association between burnout and depression, anxiety and sleep disorder in medical staff

The results of the correlation analysis between job burnout and anxiety, depression, and insomnia are shown in Table 6. MBI-EE was positively correlated with MBI-DE, PHQ-9, GAD-7, and ISI (*P* < 0.01), and negatively correlated with MBI-PA (*P* < 0.001). MBI-DE was positively correlated with MBI-EE, PHQ-9, GAD-7, and ISI (*P* < 0.01), and negatively correlated with MBI-PA (*P* < 0.01). MBI-PA was negatively correlated with MBI-EE, MBI-DE, PHQ-9, GAD-7, and ISI (*P* < 0.01) (Table 6).

**Table 6:** Correlation analysis of job burnout with anxiety, depression and insomnia.

		**PHQ-9**	**GAD-7**	**ISI**	**MBI-EE**	**MBI-DE**	**MBI-PA**
PHQ-9	*r*	1.000					
	*p*	<0.001					
GAD-7	*r*	0.810^***^	1.000				
	*p*	<0.001	<0.001				
ISI	*r*	0.657^***^	0.583^***^	1.000			
	*p*	<0.001	<0.001	<0.001			
MBI-EE	*r*	0.708^***^	0.694^***^	0.519^***^	1.000		
	*p*	<0.001	<0.001	<0.001	<0.001		
MBI-DE	*r*	0.632^***^	0.621^***^	0.396^***^	0.697^***^	1.000	
	*p*	<0.001	<0.001	<0.001	<0.001	<0.001	
MBI-PA	*r*	−0.452^***^	−0.428^***^	−0.315^***^	−0.375^***^	−0.504^***^	1.000
	*p*	<0.001	<0.001	<0.001	<0.001	<0.001	<0.001

^*^P < 0.1, ^**^P < 0.05, ^***^P < 0.01, ^****^P < 0.001.

## Discussion

This study analyzed the burnout, anxiety, depression, and sleep quality of medical staff during the COVID-19 pandemic in Shanghai. We found that medical staff had problems with job burnout, anxiety, depression, and sleep. These problems were mainly related to gender, age, length of service, education level, professional title grade, marital status, number of children, hospital level, job category, daily working time, experience working in a shelter hospital or a designated hospital for patients affected by COVID-19, and if they came from other provinces to help Shanghai. Then we made further analysis of these possible reasons, found the main influencing factors, and put forward suggestions and possible useful methods.

### The status of burnout, depression, anxiety and insomnia of medical staff during the COVID-19 in Shanghai

Our results showed that depersonalization, depression, anxiety, and insomnia scores of medical staff were significantly higher during the pandemic in Shanghai compared with norms, while lack of personal achievement scores were decreased.

Frontline medical staff have faced hard challenges during the pandemic, including an overwhelming workload and an uncertain course of the pandemic. Multiple studies have shown that frontline medical staff treating COVID-19 patients have a high risk of developing mental health conditions such as anxiety, stress, and the feeling that they are inadequately prepared for their duties (39–42). Our findings are consistent with other studies about burnout and the mental health of medical staff during COVID-19 epidemic (43). The workload of medical staff has increased significantly during the pandemic, and the sudden outbreak of the epidemic forced medical staff to perform even more high-intensity work. Due to the highly infectious nature of the mutated virus, clinical workers require higher protection specifications and hygiene standards, might create a state of tension and worry about being infected. This tension may be considered as possible factors to burnout, depression, anxiety and insomnia. The dual burden of family and job along with interprofessional work under these special circumstances may also affect medical staff burnout and mental health (44). Encouragingly, although medical staff had higher scores on scales describing depersonalization, depression, anxiety, and insomnia, emotional exhaustion scores did not increase and they had a higher sense of personal achievement than norms. This may be because medical staff are motivated by a sense of social responsibility and carry strong convictions regarding their mission to heal the sick and contribute to fighting the pandemic, and they were proud of being able to provide high-quality care and perform their duties during a pandemic (45, 46).

### Factors related to burnout, anxiety, depression, and sleep disorder

After further analysis, we found that working time, work unit, work environment and age are important influencers of burnout, depression and anxiety of medical staff during the COVID-19 in Shanghai, while they had little effect on sleep. Working hours had a positive relationship with job burnout as well as emotion. The longer the working hours, the more serious the symptoms. Medical staff with long-term work were more likely to suffer from emotional exhaustion, depersonalization and depression. In addition, the hospital levels also has certain influence on medical staff. The analysis results show that the medical staff in the tertiary hospitals have a serious lack of personal accomplishment, while the medical staff in the community hospitals and the secondary hospitals have more emotional problems, we may infer that the medical staff working in the secondary or community hospitals are more likely to have depression and anxiety.

We also found that age had a strong impact on burnout and mood among medical staff. Older medical staff were more likely to have a lack of personal accomplishment, while younger medical staff were more likely to have emotional problems such as anxiety and depression. Sulmaz et al. (12) found that medical staff over 40 were more likely to have emotional problems during COVID-19. This result was contrary to our research results, which may related to the social environment when we conducted the survey or different classification of the ages. In our study, medical staff aged from 26 to 35 and older than 45 were more likely to experience a lack of personal achievement than medical staff aged <25.

Due to the severity of the epidemic, Shanghai established many shelter hospitals or designated hospitals for COVID-19 patients during the epidemic. Therefore, we also analyzed the psychological differences between the medical staff in the shelter or COVID-19 designated hospitals and those not in the shelter hospitals. Through the results, we found that the job burnout and mental status of medical staff in the shelter hospitals or the designated hospitals for COVID-19 patients were better than those who were not in these hospitals. Medical staff who were not in the shelter hospitals or designated hospitals were more likely to have problems of emotional exhaustion, depersonalization and anxiety. The results may be related to the selection criteria of shelter hospitals or designated hospitals, those who have rich clinical experiences of the actively-signed-up medical staff may be more likely to be selected. So far, this is the first study to count and discuss the psychological differences between medical staff in the shelter or COVID-19 designated hospitals and those who were not.

Because of breakout of the epidemic, Shanghai needs the support of medical staff from all over the country during the epidemic. Therefore, we also discussed the emotional problems and differences between local medical staff in Shanghai and medical staff helping Shanghai. The results show that the emotional problems of local medical staff in Shanghai are more serious, and they are more prone to have anxiety and depression. This may be because the medical staff who help Shanghai need to provide assistance for a period of time after the middle stage of the epidemic, while the local medical staff in Shanghai have already worked at the front line from the start of the epidemic, so their job burnout and emotional symptoms are more serious than those who help Shanghai.

Finally, we analyzed associations between burnout and depression, anxiety, and sleep disorder among our participants. MBI-EE and MBI-DE were positively correlated with PHQ-9, GAD-7, and ISI, but MBI-PA was negatively correlated with MBI-EE, MBI-DE, PHQ-9, GAD-7, and ISI. Prior studies hypothesized that there is a correlation between job burnout and depression, and that the symptoms of burnout and depression partially overlap (15). Burnout was positively correlated with depression. We supplemented these findings with a correlation analysis between burnout and anxiety, which confirmed that burnout was also positively correlated with anxiety. Prior works have shown that the mental health problems of medical staff may lead to adverse events such as suicide. Approximately 13–36% of medical staff have psychological problems, but they are more reluctant to seek help than non-medical patients. The possible reasons for this may be that the consequences of seeking treatment may impact their medical career (47). We therefore must also pay attention to whether medical staff who report job burnout may also have depression or anxiety. Timely detection and intervention can effectively avoid adverse events such as suicide or self-injury. Early identification of symptoms can improve the psychological wellbeing of service providers and reduce the risk of burnout and other mental problems (48).

### Suggestion and potentially helpful methods

Job burnout, depression, anxiety and insomnia needs to be identified and addressed. We have several suggestions about these problems. The persons in charge should pay attention to the working hours of medical staff and to make appropriate adjustments which should be completed by the institution. Meanwhile, medical institution charger should pay attention to the psychological health of medical staff and give timely guidance. The problems can be solved to some extent by building a psychological assistance platform to provide psychological assistance to medical staff through the internet or face-to-face consulting service.

In addition, we should appease medical staff according to different kinds of characteristics. For example, medical staff in community hospitals and secondary hospitals have more pressures due to heavy workload, so the hospital chargers should further optimize their work and provide interventions to relieve their pressures. Adjust the working status of medical staff in tertiary hospitals and improve their personal sense of achievement by establishing a sound incentive mechanism. Paying attention to appease the emotion of younger medical staff and enhancing the sense of personal achievement of the elder medical staff may be helpful.

For the management of these problems of medical staff, some non-drug therapy were recommended such as mindfulness therapy. Several studies proved the effect of mindfulness therapy (49, 50). A study on operating room staff found that providing them with 30-min of music therapy three times a day for a consecutive month significantly decreased their emotional exhaustion, representing improved job burnout (51). In another pilot study, researchers chose mindfulness therapy based on an online app, which has also been shown to help reduce the stress and negative emotions of medical and non-medical participants, thereby improving burnout (52, 53). We therefore recommend the use of non-drug interventions such as online mindfulness therapy or music therapy to help medical staff with job burnout during the COVID-19 pandemic.

Moreover, non-invasive brain stimulation techniques (NIBS) such as transcranial magnetic stimulation (TMS) and transcranial direct current stimulation (tDCS) are also used to address anxiety, depression, and sleep (54, 55). Our prior research used functional near-infrared spectroscopy (fNIRS) to observe the efficacy of tDCS on patients with generalized anxiety disorder (GAD) and depression, reporting that anxiety, depression, and insomnia all improved after intervention and that tDCS can affect brain activity in patients with burnout (56). Another study shows that TMS can prolong patient health and reduce recurrence (57). NIBS treatment is therefore a good choice for medical workers with depression, anxiety, and insomnia. It hints that NIBS can help improve emotional and sleep problems, alleviate job burnout, and be used as long-term treatment to reduce the recurrence of negative emotions and promote emotional stability of medical staff.

## Conclusion

This study investigated the levels of burnout, depression, anxiety, and insomnia of medical staff during the COVID-19 epidemic outbreak in Shanghai. It also explored factors related to job burnout, mental health, and insomnia among medical staff. Our results showed that depersonalization, depression, anxiety, and insomnia scores were significantly higher during the pandemic compared with before, while personal achievement was higher during the pandemic. Working time, work unit, work environment and age are important influencers of burnout, depression, and anxiety of medical staff. Long working hours are the most likely causes of burnout and emotional disorders. Medical staff in primary hospitals were most likely to suffer from burnout and emotional disorders, while medical staff in tertiary hospitals had a reduced sense of personal achievement. Young medical staff are prone to negative emotions such as depression and anxiety, while older medical staff have a lower sense of personal accomplishment. For the first time, we reported that medical staff who were not in the shelter hospitals or designated hospitals were more likely to have problems of emotional exhaustion, depersonalization and anxiety than those who were in the shelter hospitals or designated hospitals. Contracting COVID-19 had no effect on medical staff. Emotional exhaustion and depersonalization were positively correlated with anxiety, depression, and sleep disorders while personal achievement was negatively correlated with these factors. In conclusion, care and supports about burnout, mental health and insomnia need to be taken to promote the mental health of medical staff. We hope that this study can provide the basis for constructing future interventions during large-scale medical emergencies and call on medical staff to actively take care of their mental health to better serve the public good.

### Limitations

There are several limitations to this survey. This was a cross-sectional survey, the results only represent the status of medical professions during the epidemic outbreak. Due to the lack of investigation on burnout, mental status and insomnia of medical staff before the outbreak of the epidemic, the norm that can be queried is too long to strongly prove the relationship between the epidemic and burnout, mental status and insomnia of medical staff. We can only infer that the outbreak of the epidemic has a negative impact on the burnout, mood and sleep of medical staff. A follow-up survey is needed to explore whether burnout, depression, anxiety, and insomnia decreased along with COVID-19 burden which can be used as evidence toward causality. In addition, as the research was conducted in an emergency, meanwhile, the research object is relatively special, we used a convenient sample in this study which made the generalization ability relatively low. Additional risk factors and their mechanisms should be investigated in further studies to comprehensively understand the mental health status of medical staff and factors that influenced it during COVID-19. Despite these limitations, this study is important for understanding medical professional mental health during the sudden onset of an epidemic and may contribute to the formulation of psychological care strategies for this important group.

## Data availability statement

The raw data supporting the conclusions of this article will be made available by the authors, without undue reservation.

## Ethics statement

The ethic review was exempted by the Ethics Committee of Yueyang Hospital of Integrated Traditional Chinese and Western Medicine.

## Author contributions

LT, Y-wW, and X-tY designed and conceptualized the study and drafted the manuscript. R-lL and W-yJ collected the information and organized the data. NZ and X-lG analyzed the data. Y-fC and W-jY supervised the whole study. All authors approved the final version of this manuscript.
